# Closed Reduction and Percutaneous Pinning versus Open Reduction and Internal Fixation in Pediatric Supracondylar Humeral Fractures: A Systematic Review

**DOI:** 10.1055/s-0045-1804496

**Published:** 2025-04-15

**Authors:** Febyan Febyan, Made Agus Maharjana, Nyoman Gede Grenata Nanda Ustriyana

**Affiliations:** 1 Departamento de Ortopedia e Traumatologia, Prof. Ngoerah General Hospital, Faculty of Medicine, Udayana University, Denpasar, Bali Surabaya, Indonésia

**Keywords:** bone nails, bone wires, elbow joint, humeral fractures, distal, meta-analysis

## Abstract

**Objective** To compare the treatment approaches of closed reduction and percutaneous pinning (CRPP) and open reduction and internal fixation (ORIF) in pediatric supracondylar humeral fractures through a systematic review of cohort and case-control studies.

**Methods** The CRPP and ORIF treatment modalities were evaluated using Flynn's functional criteria and Baumann angle as outcome measures.

**Results** The results support the clinical equivalence of CRPP ORIF regarding functional outcomes. Despite the limited number of studies and the low level of evidence of the included articles, the present study consistently reported no significant differences, which is in line with the overall results. Limited Baumann Angle data prevented conclusive comparisons in this regard. Factors such as length of hospital stay and cosmetic outcomes influence treatment decisions in pediatric supracondylar humerus fractures. A holistic approach is essential, considering clinical efficacy and patient comfort. Future research should expand the evidence base and standardize outcome assessments.

**Conclusion** Both CRPP and ORIF are viable treatments for pediatric supracondylar humerus fractures, particularly those classified as Gartland type III.

## Introduction

Supracondylar humerus fractures represent a common and clinically-significant pediatric orthopedic injury; however, ita incidence has increased in the last decades, accounting for 3% to 15% of all fractures in children. Moreover, it is the fracture that most requires surgical treatment in the pediatric population. 1 2 These fractures typically occur in children aged between 5 and 7 years, and they result from accidents such as falls on an outstretched hand. 3 4 Given the unique anatomical characteristics of pediatric bone and the potential for complications, selecting the most appropriate treatment strategy is paramount to achieve favorable outcomes.

The choice between two primary surgical techniques, closed reduction and percutaneous pinning (CRPP) and open reduction and internal fixation (ORIF), has long been a subject of debate and clinical interest. Several Brazilian authors prefer various methods of CRPP, which involves the reduction of fractured bone fragments without the need for a large incision as an advantage, followed by the insertion of percutaneous pins to maintain alignment. 1 5 6 In contrast, ORIF requires open exposure of the fracture site (a disadvantage), direct visualization, and fixation with screws or plates. Nevertheless, ORIF offers the advantage of precise anatomical reduction and potentially stronger fixation, but it comes with the drawbacks of increased surgical morbidity and extended hospitalization. 7

The decision-making process regarding the optimal treatment approach for pediatric supracondylar humerus fractures is multifaceted. It requires a comprehensive assessment of patient-specific factors, fracture characteristics, and the available evidence. Moreover, the selection of appropriate outcome measures to evaluate treatment success plays a crucial role in guiding clinical decisions. 8 Among these outcome measures, two primary parameters stand out as central to the assessment of treatment efficacy: Flynn's functional criteria and the Baumann angle. 8 9

Flynn's functional criteria provide a comprehensive evaluation of functional recovery, encompassing factors such as pain, range of motion, and strength. These criteria are particularly relevant for pediatric patients, as they reflect the restoration of elbow function, which is essential for activities of daily living and overall quality of life. 10 And the Baumann angle is a radiological measure used to assess the anatomical alignment of the elbow joint postfracture. Proper anatomical alignment is essential to minimize long-term complications, such as cubitus varus or valgus deformities. 11

While previous research has attempted to address the CRPP versus ORIF debate, inconsistencies in study designs, patient populations, and outcome measures have contributed to a lack of consensus in the field. Therefore, conducting a systematic review and meta-analysis to synthesize the existing evidence, with a specific focus on Flynn's functional criteria and the Baumann angle, is crucial. The present study aims to provide a comprehensive overview of the comparative effectiveness of CRPP and ORIF in the management of pediatric supracondylar humerus fractures. By consolidating the available data, we aim to inform clinical decision-making and contribute to the optimization of care for this prevalent and clinically-significant pediatric orthopedic condition.

## Materials and Methods

### Trial Registration

The current study was registered in the International Prospective Register of Systematic Reviews (PROSPERO) on February 14, 2024 (registration number: CRD42024506147; available from: https://www.crd.york.ac.uk/prospero/display_record.php?RecordID=506147 )

The present review employed a meta-analytic approach and adhered to the guidelines of the Preferred Reporting Items for Systematic Reviews and Meta-Analyses (PRISMA) statement, which encompass preparation, execution, and reporting stages.

### Search Strategy

We conducted a comprehensive literature search in electronic databases (PubMed/MEDLINE, ProQuest, and Cochrane Library) to identify qualified studies published between 2013 and 2013. Search terms were combined using the OR operator and linked with the AND operator. The initial search included terms such as *supracondylar humerus fractures* , *CRPP* , and *ORIF* . The subsequent search included terms such as *pediatric* , *operative* , *Flynn's criteria* , and *Baumann angle* . We also expanded the research by reviewing references, reviews, and meta-analyses; after that, we conducted a Google Scholar search to explore the citations. Then, two of the authors (MAM and FF) also screened the reference lists of the eligible articles for more potential papers. After removing duplicates, the titles and abstracts of the studies were evaluated against predetermined inclusion/exclusion criteria. Discrepancies were resolved through discussion with a third reviewer (NGGNU).

### Selection Criteria

The study focused on children with radiographically-confirmed supracondyle fractures who had undergone either ORIF or CRPP. The inclusion criteria were clinical trials conducted between 2013 and 2023 that compared ORIF and CRPP in children under 18 years of age with supracondylar humeral fractures. These trials were required to include original data on various factors such as operative time, infection, avascular necrosis, and non-union. The exclusion criteria were cases of supracondylar humeral fractures combined with other fractures, pathological fractures, as well as case reports, cadaver or model studies, and biomechanical studies. One of the authors (FF) also excluded all review studies.

### Risk of Bias Assessment

Two of the authors (MAM and FF) followed established methods using the Risk of Bias in Non-Randomized Studies – of Interventions 12 (ROBINS-I) tool to assess the risk of bias due to confounding factors, selection of participants or animals used in the study, classification of interventions, missing data, measurement of outcomes, and selection of the reported results. The tool also considers the management of incomplete outcome data, selective reporting of outcomes, and it also spots other potential biases.

For each area examined, the authors classified the risk of bias as “low,” “high,” or “unclear,” based on the available information. “Low” means there is a minimal chance that bias could affect the study's results; “high” suggests a significant chance of bias influencing the findings; and “unclear” indicates that there is not enough information to decide the level of risk of bias. 13 The authors used this approach to comprehensively assess the quality of the reviewed studies, enhancing the analyses and research conclusions by ensuring the consistent and transparent application of the ROBINS-I tool.

### Data Extraction

Two of the authors (MAM and FF) meticulously extracted pertinent data from the included articles, referencing the full-text versions. Data extraction for the current study involved a systematic approach. Pertinent data was meticulously collected from the included articles. This encompassed key characteristics of the study population, including age distribution, gender ratio (male:female), the total number of subjects, and the duration of follow-up (in months). Additionally, the Gartland classification of the patients submitted to CRPP and ORIF was extracted to assess injury severity and treatment approaches.

Regarding the outcome measures of the studies included, the authors extracted data related to Flynn's criteria, specifically evaluating the satisfactory outcomes for CRPP and ORIF. The Baumann angle, a critical indicator in the assessment of fracture reduction, was also recorded for both treatment groups. Furthermore, *p* -values were extracted to quantify the statistical significance of the differences observed between CRPP and ORIF outcomes. This meticulous data extraction process facilitated a comprehensive analysis of the study's findings and contributed to the study's overall robustness. Subsequently, the articles selected were managed using the Review Manager (RevMan, The Cochrane Collaboration, London, United Kingdom) software, version 5.4.

## Results

**Fig. 1:**
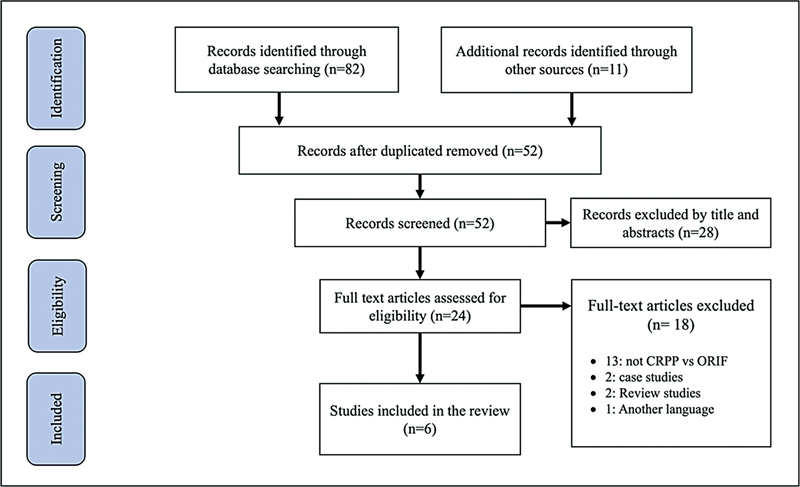
Preferred Reporting Items for Systematic Review and Meta-Analyses (PRISMA) flow diagram.

The initial literature search yielded 52 studies that could be suitable for assessment. Following a comprehensive review of titles and abstracts, 28 of these studies were excluded. Subsequently, the full-text versions of the remaining 24 studies were obtained; out of these, 18 were later disqualified. In the end, 6 studies, 14 15 16 17 18 19 involving a total of 405 subjects, satisfied the inclusion criteria for the present systematic review ( Fig. 1 ).

The authors gathered data from the selected studies to compare the functional outcomes of supracondylar humerus fractures in children after CRPP or ORIF. These studies were categorized by their level of evidence ( Table 1 ); most were cohort studies, 15 16 17 18 19 and only one was a case-control study. 14

**Table 1:** Summary of the data of the included studies

No.	Study	Journal	Study design/ level of evidence	Mean age (years)		Sex (M:F)		Number of Subjects	Mean follow-up duration (months)		Gartland classification		Flynn's criteria (satisfactory)			Mean Baumann angle	
				CRPP	ORIF	CRPP	ORIF		CRPP	ORIF	CRPP	ORIF	CRPP	ORIF	*p* -value	CRPP	ORIF
1	Tomori et al., 2018 14	*Medicine* (Baltimore)	Case-control/IV	5.4 ± 3.5	5.3 ± 2.1	10:11	6:7	CRPP: 21; ORIF: 13	7.9 ± 5.7	10.2 ± 5.3	II: 12 ; III: 9	II: 4 ; III: 9	20 (95.2%)	13 (100%)	0.83	74.9 ± 6.2	69.8 ± 3.7
2	Abousaleh et al., 2022 15	*Cureus*	Retrospective cohort/III	5.21 ± 2.17	6.69 ± 4.08	14:14	1.9:1	CRPP: 28; ORIF: 32	3.64 ± 2.61	8.13 ± 11.16	−	−	26 (92.8%)	32 (100%)	0.214	68.02 ± 9.83	70.75 ± 7.17
3	Ayub et al., 2021 16	*Journal of Pakistan Orthopaedic Association*	Retrospective cohort/III	6 ± 1.1	6.9 ± 3.83	21:9	18:12	CRPP: 30; ORIF: 30	12	12	III: 30	III: 30	30 (100%)	23 (76.6%)	<0.05	−	−
4	Dučić et al., 2016 17	*Serbian Archives of Medicine*	Cohort/III	6.7 + 1.7	6.8 + 2	29:8	23:11	CRPP: 37; ORIF: 34	−	−	II: 13 ; IV: 24	II: 17 ; III: 17	37 (100%)	31 (91.1%)	0.02	−	−
5	Hossain et al., 2023 18	*Saudi Journal of Medical and Pharmaceutical Sciences*	Cohort/III	6.5	5.9	29:14	26:11	CRPP: 43; ORIF:37	32.9 ± 12.5	29.5 ± 10.75	III: 43	III: 43	42 (97.6%)	36 (97.2%)	>0.05	−	−
6	Keskin and Sen, 2014 19	*Acta chirurgiae orthopaedicae et traumatologiae Cechoslovaca*	Cohort/II	7.04		69: 31		CRPP: 50; ORIF:50	49.2 ± 20.5	14.12 ± 12.75	−	−	47 (94%)	45 (90%)	0.774	−	−

**Abbreviations:** CRPP, closed reduction and percutaneous pinning; F, female; M, male; ORIF, open reduction and internal fixation.

The authors assessed the bias in the selected studies using the ROBINS-I tool. The method to generate random sequences was well described and presented minimal bias risk, while allocation concealment was thoroughly explained and presented a low bias risk. Blinding of the participants and personnel was not possible due to the nature of the intervention, leading to a higher bias risk. However, the outcome assessors remained unaware of the treatments, resulting in a low bias risk. Low dropout rates and reporting prespecified outcomes without selective reporting signs maintained the low bias risk. No additional bias sources were found, resulting in a low risk of bias in other areas. Overall, the current study presented a low bias risk, except for the blinding of the participants and personnel due to the intervention's nature ( Table 2 ).

**Table 2:** Risk of bias in the included studies

Study	Random sequence generation	Allocation concealment	Binding of participants and personnel	Blinding of outcome assessment	Incomplete outcome data	Selective outcome reporting	Other source of bias
Tomori et al., 2018 14	Low	Low	High	Low	Low	Low	Low
Abousaleh et al., 2022 15	Low	Low	High	Low	Low	Low	Low
Ayub et al., 2021 16	Low	Low	High	Low	Low	Low	Low
Dučić et al., 2016 17	Low	Low	High	Low	Low	Low	Low
Hossain et al., 2023 18	Low	Low	High	Low	Low	Low	Low
Keskin and Sen, 2014 19	Low	Low	High	Low	Low	Low	Low

Table 1 summarizes key characteristics from the selected studies involving a total of 405 patients. Tomori et al. 14 (2018) divided 34 patients into CRPP and ORIF groups, with follow-up durations of around 5.4 and 5.3 months respectively. Gartland classifications revealed differences between the groups. Abousaleh et al. 15 (2022) enrolled 60 patients in these groups, with CRPP having a shorter mean follow-up, of 5.21 months, and a balanced gender distribution. Ayub et al. 16 (2021) studied 60 patients in each group for 6 months, observing differences in Gartland classifications. Dučić et al. 17 (2016) included 71 patients in the same groups, with similar follow-up durations and varied Gartland classifications. Hossain et al. 18 (2023) examined 80 patients with varying follow-up durations, focusing on type-III fractures. Keskin and Sen. 19 (2014) studied 100 patients but did not report age and gender data, with the CRPP group followed up for 7.04 months. These studies collectively provide insights into treatment approaches and patient characteristics in this context.

Table 1 summarizes the outcomes of the treatment of several pediatric supracondylar humerus fractures in the studies 14 15 16 17 18 19 using Flynn's criteria and Baumann angle measurements. The CRPP and ORIF procedures generally resulted in high rates of satisfactory outcomes based on Flynn's criteria. Baumann angle measurements were comparable between the two methods across most studies, without statistically significant differences. This suggests the effectiveness of both approaches for pediatric supracondylar humerus fractures. However, variations in factors such as age, sex distribution, sample size, and follow-up duration among the studies should be considered when interpreting the results.

**Fig. 2:**
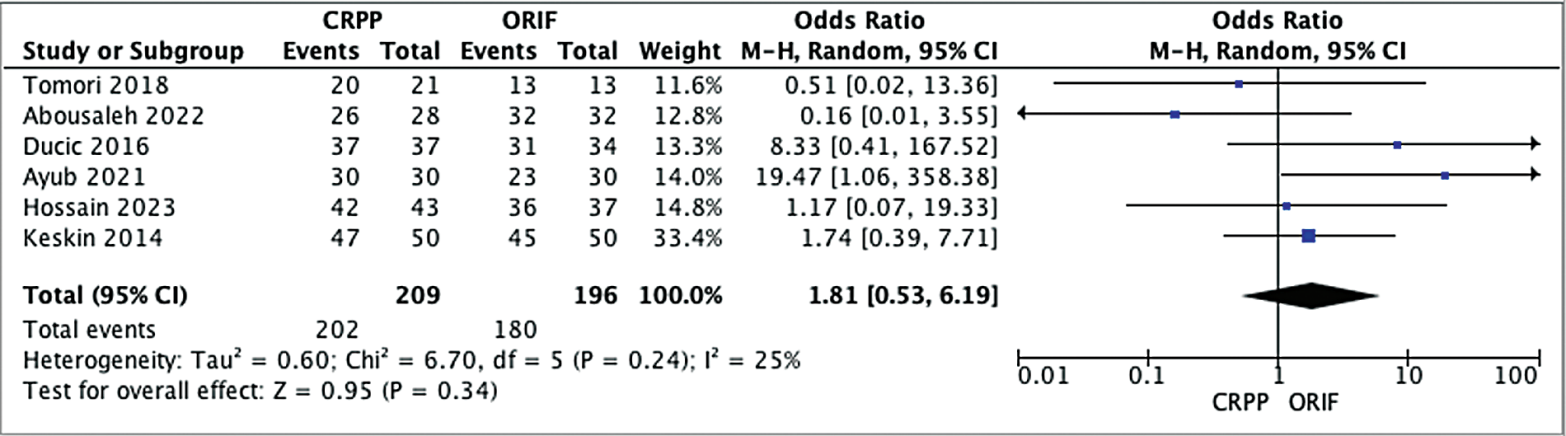
Forest plot analysis Flynn's functional criteria.

The forest plot analysis of Flynn's functional criteria in Fig. 2 suggests that there was no statistically significant difference in functional outcome between CRPP and ORIF for supracondylar humerus fractures in children. The pooled effect estimates, represented by the diamond-shaped marker, are located to the left of the line of no effect, indicating a slightly higher proportion of satisfactory outcomes in the CRPP group. However, the confidence interval for the pooled effect includes the line of no effect, indicating that the difference is not statistically significant.

**Fig. 3:**
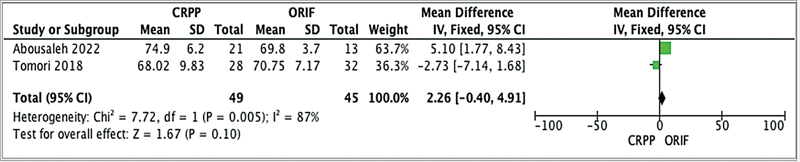
Forest plot analysis of the Baumann angle.

Flynn's functional criteria were used as one of the primary outcome measures. Fig. 3 shows that there was no statistically significant difference in terms of the Baumann angle between CRPP and ORIF ( *p* > 0.05). This suggests that both treatment approaches are viable options for supracondylar humerus fractures in children, particularly in cases of Gartland type-III fractures. The Baumann angle was also considered as a primary outcome measure. However, the limited data availability (only two studies) on the Baumann angle precluded a conclusive comparison. Therefore, no definitive conclusions regarding this measure can be drawn. Therefore, both CRPP and ORIF can be considered viable treatment options for these fractures in children.

## Discussion

Supracondylar humerus fractures pose a substantial challenge in pediatric orthopedics, demanding a nuanced approach for optimal patient outcomes. The choice between CRPP and ORIF has long been a subject of debate within the field. In this discussion, we will delve deeper into our analysis, focusing on Flynn's functional criteria while also considering Baumann angle measurements ( Figs. 2 3 ) provided by individual studies to offer a more comprehensive understanding of the comparative efficacy of these treatments. 20 21

Our analysis revealed no statistically significant difference in the outcomes according to Flynn's functional criteria between CRPP and ORIF for supracondylar humerus fractures in children ( *p* > 0.05). The findings suggest that both treatments offer comparable functional recovery, rendering them viable options, particularly for Gartland type-III fractures. The forest plot analysis visually reinforces this finding, with the pooled effect estimate slightly favoring CRPP, but within a confidence interval encompassing the line of no effect. The consistency of these results across multiple studies underscores the clinical equipoise between CRPP and ORIF concerning functional outcomes. Zhu et al. 1 reported no statistical difference in clinical outcomes between CRPP and ORIF for supracondylar fractures ( *p* > 0.05). Furthermore, Figs. 2 3 shed light on patient demographics and characteristics within each treatment group. These data underscore the diversity of our dataset, with variations in patient age, follow-up duration, and the distribution of the fractures according ot the Gartland classification. Despite these variations, the individual studies consistently report no significant disparities in functional outcomes between CRPP and ORIF, aligning harmoniously with the overall meta-analysis results.

While the Baumann angle serves as a critical radiological parameter to assess the anatomical alignment of the elbow, the limited data available on it (only two studies, 14 15 ) in the present meta-analysis hinders us from drawing definitive conclusions regarding the comparative effectiveness of CRPP and ORIF based on this parameter. Acknowledging the constraints imposed by the limited data on the Baumann angle is essential, emphasizing the need for future research with larger samples to comprehensively address this gap.

The lack of significant differences in functional outcomes between CRPP and ORIF can be attributed to several key factors, such as careful patient selection, standardized outcome assessment, surgical expertise, postoperative care, and advancements in surgical techniques. 22 23 By focusing on a relatively-homogenous group of patients with type-III fractures and employing Flynn's functional criteria as a standardized assessment tool, most of the selected studies minimized potential confounding variables and measurement bias. Additionally, the surgical teams' proficiency in both techniques likely contributed to the consistent positive outcomes in the two treatment groups. 24 25 Advances in surgical instruments, imaging, and perioperative care further improved the functional outcomes.

Besides Flynn's functional criteria and the Baumann angle, other factors warrant consideration when making treatment decisions in pediatric supracondylar humerus fractures, as seen in Figs. 2 3 . Notably, the length of hospital stay and cosmetic outcomes are aspects that can significantly influence the overall patient experience. A minimally invasive procedure, CRPP often results in shorter hospital stays than the more extensive ORIF procedure. Additionally, the limited surgical exposure and smaller incisions in CRPP may contribute to potentially superior cosmetic outcomes, a crucial consideration in the pediatric patient population. 25 26 27

In the current clinical practice, these findings empower clinicians to make treatment decisions based on individual patient characteristics and preferences. Younger children with less severe fractures may benefit from CRPP's minimally-invasive nature and shorter hospitalization, while ORIF may be preferred for cases requiring precise anatomical reduction or stronger fixation. This patient-centered approach reflects the evolving landscape of the pediatric orthopedic practice, ensuring tailored care optimizes patient outcomes and overall well-being while considering unique circumstances.

The current study presents several limitations. Firstly, the low quality of the bias of the study, which may have affected the reliability of the results. Moreover, the small number of studies included in the meta-analysis limits the statistical power to detect meaningful differences and increases the likelihood of publication bias. This restricted sample size may have led to an overestimation or underestimation of effect sizes, particularly in the presence of selective reporting or small-study effects. Further high-quality, large-scale randomized controlled trials are needed to validate these findings and provide more definitive evidence on the subject.

## Conclusion

Both CRPP and ORIF are viable treatments for pediatric supracondylar humerus fractures, particularly those classified as Gartland type-III, but further research is needed to address Baumann angle data, standardize outcome assessments, and guide treatment decisions based on functional, radiological, and cosmetic outcomes.
