# Supplementary material for: Closed Reduction and Percutaneous Pinning versus Open Reduction and Internal Fixation in Pediatric Supracondylar Humeral Fractures: A Systematic Review
Source: Rev Bras Ortop (Sao Paulo). 2025 Apr 15;60(1):s00451804496. doi: 10.1055/s-0045-1804496 (PMC12020523; doi:10.1055/s-0045-1804496)
Supplement: Related texts

# Research article: Redução fechada e fixação percutânea *versus* redução aberta e fixação interna em fraturas pediátricas supracondilares do úmero: Uma revisão sistemática

**Keywords:** articulação do cotovelo, fios ortopédicos, fraturas distais do úmero, metanálise, pinos ortopédicos

## Abstract

**Objetivo** Comparar as abordagens terapêuticas de redução fechada com fixação percutânea ( *closed reduction and percutaneous pinning* , CRPP, em inglês) e redução aberta com fixação interna ( *open reduction and internal fixation* , ORIF, em inglês) em fraturas supracondilares do úmero em pacientes pediátricos por meio de uma revisão sistemática de estudos de coorte e caso-controle.

**Métodos** As modalidades de tratamento CRPP e ORIF foram avaliadas usando os critérios funcionais de Flynn e o ângulo de Baumann como medidas de desfechos.

**Resultados** Os resultados deste estudo apoiam a equivalência clínica da CRPP e da ORIF em relação aos desfechos funcionais. Apesar do número limitado de estudos e do baixo nível de evidência dos artigos incluídos, este estudo não relatou diferenças significativas, o que condiz com os resultados gerais. Dados limitados sobre o ângulo de Baumann impediram comparações conclusivas a este respeito. Fatores como duração da internação hospitalar e resultados estéticos influenciaram as decisões de tratamento em fraturas supracondilares do úmero em pacientes pediátricos. Uma abordagem holística é essencial, considerando tanto a eficácia clínica quanto o conforto do paciente. Pesquisas futuras devem expandir a base de evidências e padronizar as avaliações de desfechos.

**Conclusão** A CRPP e a ORIF são tratamentos viáveis para fraturas supracondilares do úmero em pacientes pediátricos, particularmente as classificadas como tipo III de Gartland.

## Introdução

As fraturas supracondilares do úmero são lesões ortopédicas pediátricas comuns e clinicamente significativas. No entanto, sua incidência aumentou nas últimas décadas, e elas representam entre 3% e 15% de todas as fraturas em crianças. Além disso, são as fraturas que mais requerem tratamento cirúrgico na população pediátrica; 1 2 geralmente ocorrem em crianças de 5 a 7 anos e são decorrentes de acidentes, como quedas sobre a mão estendida. 3 4 Dadas as características anatômicas únicas do osso pediátrico e a possibilidade de complicações, a escolha da estratégia terapêutica mais apropriada é fundamental para alcançar desfechos favoráveis.

A escolha entre duas técnicas cirúrgicas primárias, a redução fechada e fixação percutânea ( *closed reduction and percutaneous pinning* , CRPP, em inglês) e a redução aberta e fixação interna ( *open reduction and internal fixation* ORIF, em inglês), há muito é assunto de debate e interesse clínico. Vários autores brasileiros preferem diversos métodos de CRPP, que consiste na redução de fragmentos ósseos fraturados sem a necessidade de incisão extensa (uma vantagem da técnica), seguida da inserção de pinos percutâneos para a manutenção do alinhamento. 1 5 6 Por outro lado, a ORIF requer exposição aberta do sítio da fratura (uma desvantagem), visualização direta e fixação com parafusos ou placas. No entanto, a ORIF tem como vantagem a redução anatômica precisa e fixação maior, mas suas desvantagens também incluem maior morbidade cirúrgica e hospitalização prolongada. 7

O processo de tomada de decisão sobre a abordagem terapêutica ideal para fraturas supracondilares do úmero em pacientes pediátricos é multifacetado. Faz-se necessária uma avaliação abrangente dos fatores específicos do paciente, das características da fratura e das evidências disponíveis. Além disso, a seleção de medidas de desfechos apropriadas para a avaliação do sucesso do tratamento desempenha um papel crucial na orientação das decisões clínicas. 8 Entre essas medidas de desfechos, dois parâmetros primários se destacam como centrais para a avaliação da eficácia do tratamento: os critérios funcionais de Flynn e o ângulo de Baumann. 8 9

Os critérios funcionais de Flynn permitem uma avaliação abrangente da recuperação funcional, e abrangem fatores como dor, amplitude de movimento e força. Esses critérios são particularmente relevantes em pacientes pediátricos, pois refletem a restauração da função do cotovelo, que é essencial para as atividades da vida diária e qualidade de vida geral. 10 Enquanto isso, o ângulo de Baumann é uma medida radiológica usada para a avaliação do alinhamento anatômico da articulação do cotovelo após a fratura. O alinhamento anatômico adequado é essencial para minimizar complicações de longo prazo, como deformidades cubitais em varo ou valgo. 11

Embora pesquisas anteriores tenham tentado abordar o debate sobre CRPP ou ORIF, inconsistências em delineamentos experimentais, amostras e medidas de desfechos contribuíram para a ausência de consenso na área. Portanto, uma revisão sistemática e metanálise para sintetizar as evidências existentes, centrada especificamente nos critérios funcionais de Flynn e no ângulo de Baumann, é crucial. Este estudo visa dar uma visão geral abrangente da eficácia comparativa da CRPP e da ORIF no tratamento de fraturas supracondilares do úmero em pacientes pediátricos. Ao consolidar os dados disponíveis, pretendemos dar elementos para a tomada de decisão clínica e contribuir para a otimização do tratamento desta doença ortopédica pediátrica prevalente e clinicamente significativa.

## Materiais e Métodos

## Registro do Estudo

Este estudo foi registrado no International Prospective Register of Systematic Reviews (PROSPERO) em 14 de fevereiro de 2024 (número de registro: CRD42024506147; disponível em: https://www.crd.york.ac.uk/prospero/display_record.php?RecordID=506147 )

Este estudo de revisão empregou uma abordagem metanalítica e aderiu às diretrizes da declaração Preferred Reporting Items for Systematic Reviews and Meta-Analyses (PRISMA), que abrange as fases de preparação, execução e relato.

## Estratégia de Busca

Realizamos uma busca bibliográfica abrangente em bancos de dados eletrônicos (PubMed/MEDLINE, ProQuest e Cochrane Library) para identificar estudos de qualidade publicados entre 2013 e 2013. Os termos de busca foram combinados com o operador OR (“OU”) e vinculados com o operador AND (“E”). A busca inicial incluiu termos como *supracondylar humerus fractures* (“fraturas supracondilares do úmero”), *CRPP* e *ORIF* . A busca subsequente incluiu termos como *pediatric* (“pediátrico”), *operative* (“operatório”), *Flynn's criteria* (“critérios de Flynn”) e *Baumann angle* (“ângulo de Baumann”). Também expandimos a pesquisa ao revisar referências, revisões e metanálises, e, então, fizemos uma busca no Google Acadêmico para explorar as citações. Em seguida, dois dos autores (MAM e FF) também examinaram as listas de referências dos artigos elegíveis para o possível acréscimo de artigos. Após a remoção de duplicatas, os títulos e resumos dos estudos foram avaliados em relação aos critérios de inclusão/exclusão predeterminados. As discrepâncias foram resolvidas por meio de discussão com um terceiro revisor (NGGNU).

## Critérios de Seleção

O estudo se centrou em crianças com fraturas supracondilares confirmadas radiograficamente que foram submetidas a ORIF ou CRPP. Os critérios de inclusão foram estudos clínicos conduzidos entre 2013 e 2023 que compararam ORIF e CRPP em crianças menores de 18 anos com fraturas supracondilares do úmero. Esses estudos tinham de incluir dados originais sobre vários fatores, como tempo operatório, infecção, necrose avascular e ausência de consolidação. Os critérios de exclusão foram casos de fraturas supracondilares do úmero combinadas com outras fraturas, fraturas patológicas e relatos de casos, estudos em cadáveres ou modelos e estudos biomecânicos. Um dos autores (FF) também excluiu os estudos de revisão.

## Avaliação do Risco de Viés

Dois dos autores (MAM e FF) seguiram métodos estabelecidos pela ferramenta Risk of Bias in Non-Randomized Studies – of Interventions (ROBINS-I) para avaliar os riscos de viés 12 devido a fatores de confusão, seleção de participantes ou animais usados no estudo, classificação de intervenções, ausência de dados, medidas de desfecho e seleção dos resultados relatados. A ferramenta também considera o manejo de dados de resultados incompletos, o relato seletivo de desfechos e determina outros possíveis vieses.

Os riscos de viés de cada área examinada foram classificados pelos autores como “baixo”, “alto” ou “pouco claro”, com base nas informações disponíveis. “Baixo” significa que há uma chance mínima de que o viés possa afetar os resultados do estudo; “alto” sugere uma chance significativa de o viés influenciar os achados; e “pouco claro” indica a ausência de informações suficientes para decidir o nível de risco de viés. 13 Os autores usaram essa abordagem para avaliar de forma abrangente a qualidade dos estudos revisados, o que aprimorou as análises e conclusões da pesquisa ao assegurar a aplicação consistente e transparente da ferramenta ROBINS-I.

## Extração de Dados

Dois dos autores (MAM e FF) extraíram meticulosamente dados pertinentes das versões completas dos artigos incluídos. A extração de dados foi feita com uma abordagem sistemática. Os dados pertinentes foram meticulosamente coletados, incluindo as características principais da população, como distribuição etária, razão de sexo (masculino:feminino), número total de indivíduos e duração do acompanhamento (em meses). Além disso, a classificação de Gartland dos indivíduos submetidos a CRPP ou ORIF foi extraída para a avaliação da gravidade da lesão e das abordagens terapêuticas.

Em relação às medidas de desfecho dos estudos, os autores extraíram dados relacionados aos critérios de Flynn e avaliaram especificamente os desfechos satisfatórios da CRPP e da ORIF. O ângulo de Baumann, um indicador crítico na avaliação da redução de fraturas, também foi registrado em ambos os grupos de tratamento. Além disso, os valores de *p* foram extraídos para quantificar a significância estatística das diferenças observadas entre os desfechos de CRPP e ORIF. Esse meticuloso processo de extração de dados facilitou uma análise abrangente dos achados e contribuiu para a robustez geral do estudo. Posteriormente, os artigos selecionados foram analisados com o programa Review Manager (RevMan, The Cochrane Collaboration, Londres, Reino Unido), versão 5.4.

## Resultados

A busca bibliográfica inicial rendeu 52 estudos que poderiam ser adequados para avaliação. Após uma revisão abrangente de títulos e resumos, 28 desses estudos foram excluídos. Posteriormente, as versões em texto completo dos 24 estudos restantes foram obtidas, e 18 artigos foram desqualificados. No final, 6 estudos 14 15 16 17 18 19 satisfizeram os critérios de inclusão desta revisão sistemática ( Fig. 1 ).

**Fig. 1:** Fluxograma da declaração Preferred Reporting Items for Systematic Review and Meta-Analyses (PRISMA). **Abreviaturas:** CRPP, *closed reduction and percutaneous pinning* (redução fechada e fixação percutânea); ORIF, *open reduction and internal fixation* (redução aberta e fixação interna).

Os autores reuniram os dados dos estudos escolhidos para a comparação dos desfechos funcionais de fraturas supracondilares no úmero de crianças após CRPP ou ORIF. Esses estudos foram categorizados por seu nível de evidência ( Tabela 1 ); a maioria 15 16 17 18 19 era estudos de coorte, e somente um estudo 14 era de caso-controle.

**Tabela 1:** Resumo dos dados dos estudos incluídos

Número	Estudo	Publicação	Delineamento experimental/nível de evidência	média de idade (anos)		Sexo (M:F)		Número de indivíduos	Média do tempo de acompanhamento (meses)		Classificação de Gartland		Critérios de Flynn (satisfatórios)			Média do ângulo de Baumann	
				CRPP	ORIF	CRPP	ORIF		CRPP	ORIF	CRPP	ORIF	CRPP	ORIF	Valor de *p*	CRPP	ORIF
1	Tomori et al., 2018 14	*Medicine* (Baltimore)	Caso-controle/IV	5,4 ± 3,5	5,3 ± 2,1	10:11	6:7	CRRP: 21 ORIF: 13	7,9 ± 5,7	10,2 ± 5,3	II: 12 ; III: 9	II: 4 ; III: 9	20 (95,2%)	13 (100%)	0,83	74,9 ± 6,2	69,8 ± 3,7
2	Abousaleh et al., 2022 15	*Cureus*	Coorte retrospectiva/III	5,21 ± 2,17	6,69 ± 4,08	14:14	1,9:1	CRRP: 28 ORIF: 32	3,64 ± 2,61	8,13 ± 11,16	−	−	26 (92,8%)	32 (100%)	0,214	68,02 ± 9,83	70,75 ± 7,17
3	Ayub et al., 2021 16	*Journal of Pakistan Orthopaedic Association*	Coorte retrospectiva/III	6 ± 1,1	6,9 ± 3,83	21:9	18:12	CRRP: 30 ORIF: 30	12	12	III: 30	III: 30	30 (100%)	23 (76,6%)	<0,05	−	−
4	Dučić et al., 2016 17	*Serbian Archives of Medicine*	Coorte/III	6,7 + 1,7	6,8 + 2	29:8	23:11	CRRP: 37 ORIF: 34	−	−	II: 13; IV: 24	II: 17; III: 17	37 (100%)	31 (91,1%)	0,02	−	−
5	Hossain et al., 2023 18	*Saudi Journal of Medical and Pharmaceutical Sciences*	Coorte/III	6,5	5,9	29:14	26:11	CRRP: 43 ORIF:37	32,9 ± 12,5	29,5 ± 10,75	III: 43	III: 43	42 (97,6%)	36 (97,2%)	>0,05	−	−
6	Keskin e Sen, 2014 19	*Acta chirurgiae orthopaedicae et traumatologiae Cechoslovaca*	Coorte/II	7,04		69: 31		CRRP: 50 ORIF:50	49,2 ± 20,5	14,12 ± 12,75	−	−	47 (94%)	45 (90%)	0,774	−	−

**Abreviaturas:** CRPP, *closed reduction and percutaneous pinning* (redução fechada e fixação percutânea); F, feminino; M, masculino; ORIF, *open reduction and internal fixation* (redução aberta e fixação interna).

Os autores avaliaram o viés nos estudos escolhidos usando a ferramenta ROBINS-I. O método para a geração de sequências aleatórias foi bem descrito e teve risco mínimo de viés; a ocultação de alocação foi explicada por completo e teve baixo risco de viés. O cegamento dos participantes e da equipe não foi possível devido à natureza da intervenção, o que acarretou um maior risco de viés. No entanto, os avaliadores dos desfechos desconheciam os tratamentos, o que resultou em baixo risco de viés. Baixas taxas de abandono e relatos de desfechos pré-especificados sem sinais de seletividade mantiveram o risco de viés baixo. Nenhuma outra fonte de viés foi encontrada, o que gerou um baixo risco de viés em outras áreas. De modo geral, este estudo apresentou risco de viés baixo, exceto pelo cegamento dos participantes e da equipe devido à natureza da intervenção ( Tabela 2 ).

**Tabela 2:** Avaliação do risco de viés dos estudos incluídos

Estudo	Geração de sequência aleatória	Ocultação da alocação	Cegamento de participantes e equipe	Cegamento da avaliação de desfecho	Desfechos com dados incompletos	Relato seletivo de desfechos	Outras fontes de viés
Tomori et al., 2018 14	Baixo	Baixo	Alto	Baixo	Baixo	Baixo	Baixo
Abousaleh et al., 2022 15	Baixo	Baixo	Alto	Baixo	Baixo	Baixo	Baixo
Ayub et al., 2021 16	Baixo	Baixo	Alto	Baixo	Baixo	Baixo	Baixo
Dučić et al., 2016 17	Baixo	Baixo	Alto	Baixo	Baixo	Baixo	Baixo
Hossain et al., 2023 18	Baixo	Baixo	Alto	Baixo	Baixo	Baixo	Baixo
Keskin e Sen, 2014 19	Baixo	Baixo	Alto	Baixo	Baixo	Baixo	Baixo

A Tabela 1 resume as principais características dos estudos selecionados com um total de 405 pacientes. Tomori et al. 14 (2018) dividiram 34 pacientes em grupos de CRPP e ORIF, com durações de acompanhamento de cerca de 5,4 e 5,3 meses, respectivamente. As classificações de Gartland revelaram diferenças entre os grupos. Abousaleh et al. 15 (2022) incluíram 60 pacientes nesses grupos; o grupo CRPP teve acompanhamento médio menor, de 5,21 meses, e uma distribuição de gênero equilibrada. Ayub et al. 16 (2021) estudaram 60 pacientes durante 6 meses em cada grupo, e observaram diferenças nas classificações de Gartland. Dučić et al. 17 (2016) incluíram 71 pacientes submetidos a CRPP ou ORIF com durações de acompanhamento semelhantes e classificações de Gartland variadas. Hossain et al. 18 (2023) examinaram 80 pacientes com durações de acompanhamento variadas e foco em fraturas do tipo III. Keskin e Sen. 19 (2014) estudaram 100 pacientes submetidos a CRPP e acompanhados por 7,04 meses, mas não relataram detalhes de idade e gênero. Esses estudos coletivamente forneceram informações relevantes sobre as abordagens terapêuticas e as características dos pacientes.

A Tabela 1 resume os desfechos de várias fraturas supracondilares do úmero em pacientes pediátricos nos estudos 14 15 16 17 18 19 usando os critérios de Flynn e as medidas do ângulo de Baumann. A tabela revela que a CRPP e a ORIF geralmente apresentam altas taxas de resultados satisfatórios com base nos critérios de Flynn. As medidas do ângulo de Baumann foram comparáveis entre os dois métodos na maioria dos estudos, sem diferenças estatisticamente significativas. Isso sugere a eficácia das duas abordagens em fraturas supracondilares do úmero em pacientes pediátricos. No entanto, variações em fatores como idade, distribuição dos pacientes por sexo, número de indivíduos e duração do acompanhamento entre os estudos devem ser consideradas na interpretação dos resultados.

A análise em gráfico de floresta na Fig. 2 dos critérios funcionais de Flynn sugere que não há diferença estatisticamente significativa no desfecho funcional de CRPP e ORIF em fraturas supracondilares do úmero em crianças. As estimativas de efeito agrupado, representadas pelo marcador em forma de diamante, estão à esquerda da linha de efeito nulo, o que indica uma proporção ligeiramente maior de desfechos satisfatórios no grupo CRPP. No entanto, o intervalo de confiança do efeito agrupado inclui a linha de efeito nulo, o que indica que a diferença não é estatisticamente significativa.

**Fig. 2:** Análise em gráfico de floresta dos critérios funcionais de Flynn. **Abreviaturas:** CRPP, *closed reduction and percutaneous pinning* (redução fechada e fixação percutânea); df, *degrees of freedom* (graus de liberdade); IC, intervalo de confiança; ORIF, *open reduction and internal fixation* (redução aberta e fixação interna).

Os critérios funcionais de Flynn foram usados como uma das medidas primárias de desfechos. A análise da Fig. 3 mostra que não houve diferença estatisticamente significativa quanto ao ângulo de Baumann entre CRPP e ORIF ( *p* > 0,05). Isso sugere que as duas abordagens terapêuticas são opções viáveis em fraturas supracondilares do úmero em crianças, particularmente em casos de fraturas classificadas como de tipo III de Gartland. O ângulo de Baumann também foi considerado uma medida primária de desfecho. No entanto, a disponibilidade limitada de dados (apenas dois estudos) sobre o ângulo de Baumann impediu uma comparação conclusiva. Portanto, tanto CRPP quanto ORIF podem ser consideradas opções de tratamento viáveis para essas fraturas em crianças.

**Fig. 3:** Análise em gráfico de floresta do ângulo de Baumann. **Abreviaturas:** CRPP, *closed reduction and percutaneous pinning* (redução fechada e fixação percutânea); DP, desvio padrão; df, *degrees of freedom* (graus de liberdade); IC, intervalo de confiança; IV, ponderação; ORIF, *open reduction and internal fixation* (redução aberta e fixação interna).

## Discussão

As fraturas supracondilares do úmero representam um desafio substancial na ortopedia pediátrica, pois exigem uma abordagem matizada para a obtenção dos desfechos ideais para o paciente. A escolha entre CRPP e ORIF há muito é assunto de debate na área. Nesta discussão, nos aprofundaremos em nossa análise, centrando-nos nos critérios funcionais de Flynn e nas medidas do ângulo de Baumann ( Figs. 2 3 ) fornecidos pelos estudos para maior compreensão da eficácia comparativa desses tratamentos. 20 21

Nossa análise não revelou diferença estatisticamente significativa entre CRPP e ORIF quanto aos desfechos, segundo os critérios funcionais de Flynn, para fraturas supracondilares do úmero em crianças ( *p* > 0,05). Os achados sugerem que ambas as modalidades terapêuticas resultam em recuperação funcional comparável, o que as torna opções viáveis, particularmente para o tratamento de fraturas de tipo III de Gartland. A análise do gráfico de floresta reforça visualmente esse achado, com a estimativa do efeito combinado favorecendo ligeiramente a CRPP, mas dentro de um intervalo de confiança que abrange a linha de efeito nulo. A consistência desses resultados em diversos estudos ressalta o equilíbrio clínico entre CRPP e ORIF em relação aos desfechos funcionais. Zhu et al. 1 não relataram diferença estatística no resultado clínico entre CRPP e ORIF em fraturas supracondilares ( *p* > 0,05). Além disso, as Figs. 2 3 esclarecem a demografia e as características dos pacientes de cada grupo terapêutico. Esses dados ressaltam a diversidade do nosso conjunto de dados, com variações na idade dos pacientes, na duração do acompanhamento e na distribuição das fraturas segundo a classificação de Gartland. Apesar dessas variações, os estudos individuais consistentemente não relatam disparidades significativas nos desfechos funcionais entre CRPP e ORIF, e se alinham harmoniosamente aos resultados gerais da metanálise.

Embora o ângulo de Baumann seja um parâmetro radiológico importante para a avaliação do alinhamento anatômico do cotovelo, os dados limitados sobre isso (somente dois estudos 14 15 ) disponíveis em nossa metanálise nos impedem de tirar conclusões definitivas sobre a eficácia comparativa de CRPP e ORIF com base neste parâmetro. Reconhecer as restrições impostas pelos dados limitados sobre o ângulo de Baumann é essencial, e enfatiza a necessidade de pesquisas futuras com tamanhos maiores de amostra para abordar melhor essa lacuna.

A ausência de diferenças significativas nos desfechos funcionais entre CRPP e ORIF no tratamento de fraturas supracondilares do úmero em pacientes pediátricos pode ser atribuída a diversos fatores essenciais, como a seleção cuidadosa dos pacientes, a avaliação padronizada dos desfechos, a perícia cirúrgica, os cuidados pós-operatórios e os avanços nas técnicas cirúrgicas. 22 23 Ao se centrar em um grupo relativamente homogêneo de pacientes com fraturas de tipo III e empregar os critérios funcionais de Flynn como uma ferramenta de avaliação padronizada, a maioria dos estudos selecionados em nossa análise minimizou possíveis variáveis de confusão e vieses de medição. Além disso, a proficiência das equipes cirúrgicas nas técnicas de CRPP e ORIF provavelmente contribuiu para desfechos positivos consistentes em ambos os grupos de tratamento. 24 25 Avanços em instrumentos cirúrgicos, imagens e cuidados perioperatórios melhoraram ainda mais os desfechos funcionais.

Além dos critérios funcionais de Flynn e do ângulo de Baumann, outros fatores devem ser considerados nas decisões de tratamento de fraturas supracondilares do úmero em pacientes pediátricos, como se pode ver nas Figs. 2 3 . De modo notável, a duração das internações hospitalares e os desfechos estéticos são aspectos que podem influenciar significativamente a experiência geral do paciente. A CRPP, um procedimento minimamente invasivo, é geralmente associada a internações hospitalares mais curtas do que o procedimento de ORIF, que é mais extenso. Ademais, a exposição cirúrgica limitada e as incisões menores da CRPP podem contribuir para desfechos estéticos superiores, uma consideração crucial na população de pacientes pediátricos. 25 26 27

Na prática clínica atual, esses achados capacitam os médicos a tomar decisões terapêuticas com base nas características e preferências individuais do paciente. Crianças mais novas com fraturas menos graves podem ser beneficiadas pela natureza minimamente invasiva e da hospitalização mais curta da CRPP, ao passo a ORIF pode ser preferida nos casos que exigem redução anatômica precisa ou fixação mais robusta. Essa abordagem centrada no paciente reflete o cenário em evolução da prática ortopédica pediátrica, e garante que o atendimento personalizado otimize os desfechos do paciente e seu bem-estar geral ao mesmo tempo em que considera circunstâncias únicas.

Este estudo tem várias limitações. Em primeiro lugar, a baixa qualidade de viés, que pode ter afetado a confiabilidade dos resultados. Além disso, o pequeno número de estudos incluídos na meta-análise limita o poder estatístico para a detecção de diferenças significativas e aumenta a probabilidade de viés de publicação. Este tamanho de amostra restrito pode ter levado a uma superestimação ou subestimação dos tamanhos do efeito, particularmente na presença de relatos seletivos ou efeitos de pequenos estudos. Mais ensaios clínicos randomizados de alta qualidade e em larga escala são necessários para validar esses achados e fornecer evidências mais definitivas sobre o assunto.

## Conclusão

Tanto a CRPP quanto a ORIF são tratamentos viáveis em fraturas supracondilares do úmero em pacientes pediátricos, particularmente as de tipo III de Gartland, mas mais pesquisas são necessárias para abordar os dados sobre o ângulo de Baumann, padronizar as avaliações de desfechos e orientar as decisões terapêuticas com base em desfechos funcionais, radiológicos e cosméticos.
